# Enhancing the Mechanical Toughness of Epoxy-Resin Composites Using Natural Silk Reinforcements

**DOI:** 10.1038/s41598-017-11919-1

**Published:** 2017-09-20

**Authors:** Kang Yang, Sujun Wu, Juan Guan, Zhengzhong Shao, Robert O. Ritchie

**Affiliations:** 1 Intl. Research Center for Advanced Structural and Biomaterials, School of Materials Science and Engineering, Beihang University, Beijing, 100191 China; 2 State Key Laboratory of Molecular Engineering of Polymers, Laboratory of Advanced Materials, Department of Macromolecular Science, Fudan University, Shanghai, 200433 China; 3 Materials Sciences Division, Lawrence Berkeley National Laboratory and Department of Materials Science & Engineering, University of California, Berkeley, CA94720 USA

## Abstract

Strong and tough epoxy composites are developed using a less-studied fibre reinforcement, that of natural silk. Two common but structurally distinct silks from the domestic *B. mori/Bm* and the wild *A. pernyi/Ap* silkworms are selected in fabric forms. We show that the toughening effects on silk-epoxy composites or SFRPs are dependent on the silk species and the volume fraction of silk. Both silks enhance the room-temperature tensile and flexural mechanical properties of the composite, whereas the more resilient *Ap* silk shows a more pronounced toughening effect and a lower critical reinforcement volume for the brittle-ductile transition. Specifically, our 60 vol.% *Ap*-SFRP displays a three-fold elevation in tensile and flexural strength, as compared to pure epoxy resin, with an order of magnitude higher breaking energy via a distinct, ductile failure mode. Importantly, the 60 vol.% *Ap*-SFRP remains ductile with 7% flexural elongation at lower temperatures (−50 °C). Under impact, these SFRPs show significantly improved energy absorption, and the 60 vol.% *Ap*-SFRP has an impact strength some eight times that of pure epoxy resin. The findings demonstrate both marked toughening and strengthening effects for epoxy composites from natural silk reinforcements, which presents opportunities for mechanically superior and “green” structural composites.

## Introduction

Epoxy resin is used extensively as the matrix in high-performance polymer composites for aeronautical and astronautical applications. Carbon fibre (CF), as one of the stiffest and strongest fibre reinforcements, is commonly selected to reinforce such epoxy matrices. However, one critical problem for these safety-critical applications is the brittle mechanical performance and poor toughness of CF reinforced epoxy composites, especially under high-rate/impact loading and at low temperatures^1–4^. Accordingly, there has been a concerted effort in recent years to improve the impact and low-temperature toughness of such epoxy-based composites^5–7^. “Hard” nano-fillers such as graphene nano-sheets carbon nanotubes are commonly used to enhance the low-temperature toughness of polymer composites^8–12^. Alternatively, “soft” rubbery materials have been used to improve the low-temperature toughness of epoxy-resin composites^13,14^. However, to date there have been few attempts to improve the impact and low-temperature toughness of these composites through modification of the fibre reinforcement.

**Figure 1:**
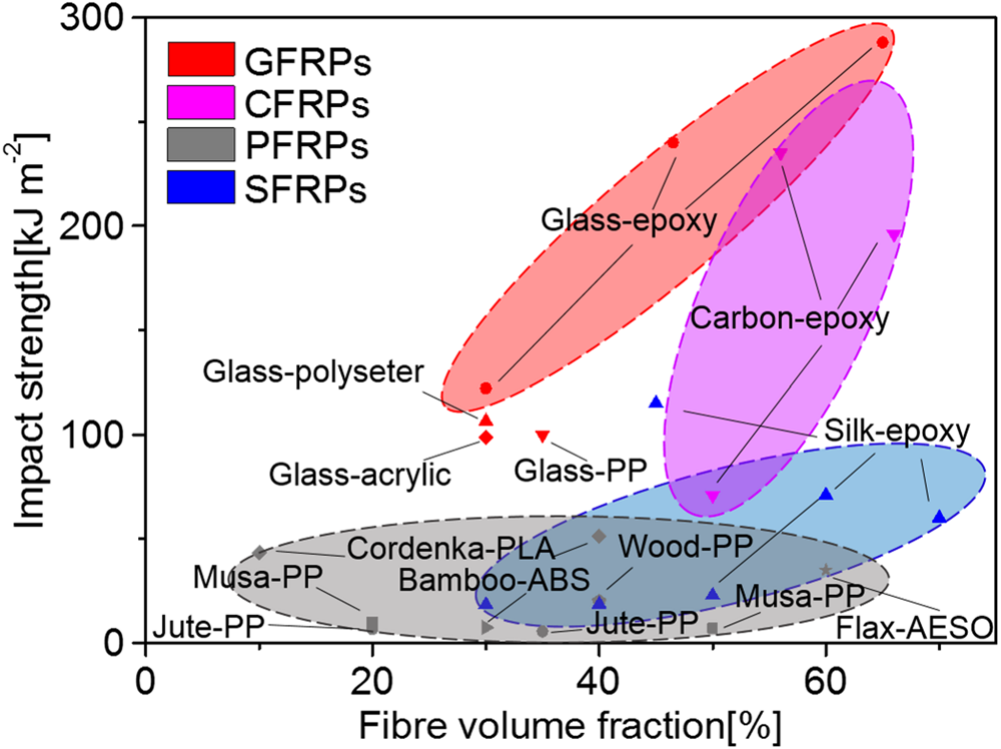
Comparison of the impact strength at room temperature of polymer composites reinforced by glass fibres, carbon fibres, plant fibres and silk fibres. The corresponding composites are respectively abbreviated as GFRP (Glass Fibre Reinforced Plastics), CFRP (Carbon Fibre Reinforced Plastics), PFRP (Plant Fibre Reinforced Plastics) and SFRP (Silk Fibre Reinforced Plastics).

Impact strength is one important measure of material’s mechanical toughness or resistance to fracture^15–17^. As polymer composites are increasingly finding applications, much work has focused on identifying their fundamental toughening mechanisms^18,19^. Figure 1 presents the impact strength, measured with Charpy impact tests, of a wide range of popular, representative polymer composites as a function of the reinforcement volume fraction. What is striking about this comparison is that many widely-used high-performance synthetic fibre reinforcements, such as UHMWPE (ultra-high molecular weight polyethylene) and natural fibres, *e.g*., flax fibre, do not impart high impact strength at all, despite their high strength and stiffness^20–23^. Carbon, glass and silk fibres can lead to impact strengths greater than 100 kJ m^−2^ in epoxy-resin matrix composites but of these fibres, silk fibres are the only natural reinforcements to provide such high impact strength. In this regard, the impact strength of polymer composites reinforced by plant fibres does not seem to be dependent on the volume fraction, whereas glass fibre and carbon fibre composites show a clear dependency on reinforcement volume fraction. Consequently, it is important to seek a complete mechanistic understanding of the origins of the impact-resistant behaviour of many of these fibre-reinforced composites, particularly in terms of the effect of reinforcement volume fraction.

Silk, traditionally as a high-profile textile fibre and now as a versatile protein material, has been popularly utilized for use in biomaterials, electronic and optical devices, as well as for structural composites^24–27^. Akin to most natural materials, the structural morphology of natural silk fibres is hierarchical, with their macro mechanical properties originating from their nano-fibrillar and semi-crystalline nanostructures^28,29^. Reported values of the toughness, or breaking energy, of silks, measured in uniaxial tensile stress-strain tests, range from 60 to 80 MJ.m^−3^ depending on the different fibre types and other factors such as degree of hydration^30–33^. In particular, spider dragline silks have been found to display enhanced modulus and strength and thus enhanced toughness, at cryogenic temperatures, specifically with a breaking energy of 214 MJ.m^−3^ under ambient conditions^34,35^. Moreover, spider silks and silkworm silks have both been found in bullet-impact experiments to generate significant elastic and plastic deformation at high strain rates to create good impact toughness^36,37^. Silk as reinforcement fibre has also been shown to improve the tensile and flexural strengths of epoxy composites^38^. However, the use of strong and tough fibres, as opposed to strong and stiff fibres, to reinforce polymer-matrix composites has never been fully explored in detail. Accordingly, the rationale for this work is to explore the use of natural silk fibre reinforcements in epoxy-resin composites to generate high toughness, specifically under low temperature and impact conditions.

## Results

### Microstructure and mechanical properties of silk-epoxy composites at ambient temperatures

**Figure 2:**
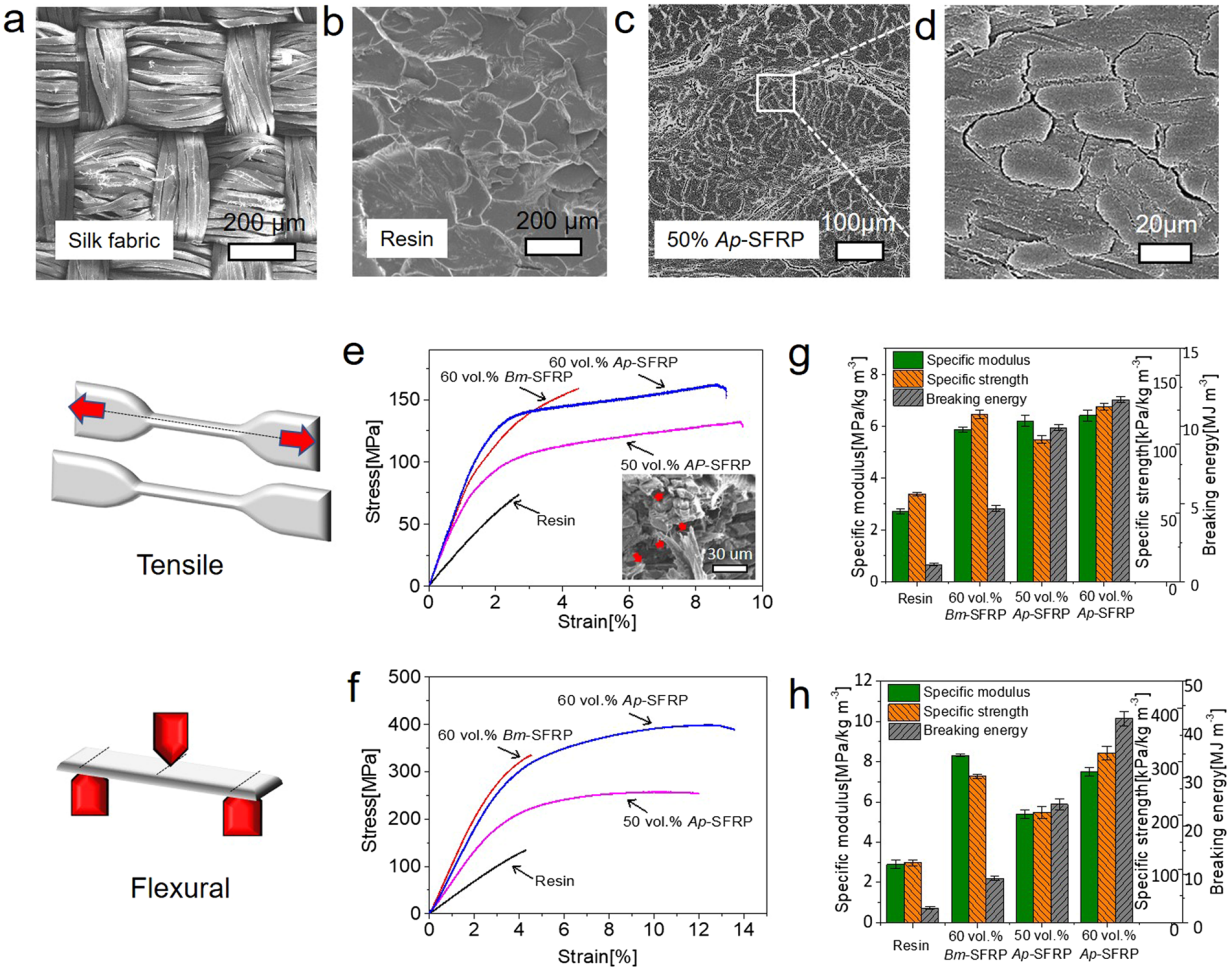
Scanning electron microscopy (SEM) images of the microstructure of (**a**) the plain woven *Ap* silk fabric, (**b**) pure epoxy resin, (**c**) the fabricated silk fibre-reinforced composites (SFRP) and (**d**) an enlarged view of the local region in (**c**). Comparison of stress-strain curves of pure epoxy resin, 60 vol.%-*Bm*-SFRP composite and 50/60 vol.%-*Ap*-SFRP composite at quasi-static strain rates under ambient conditions (temperature ~20 °C, humidity ~50%) under uniaxial tensile mode (**e**) and flexural mode (**f**). The inset in (**e**) shows the fracture morphology of 50 vol.% *Ap*-SFRP with arrows denoting the fractured and pulled-out fibres. (**g**) and (**h**) show derived mechanical properties at room temperature under quasi-static strain rates including specific modulus (refer to left axis), specific strength (refer to right inside axis) and breaking energy as a measure of toughness (refer to right outside axis).

*B. mori*/*Bm* silk and *A. pernyi*/*Ap* silk are the two common natural silks used for textile fabrics. We incorporate silk fabrics at 30% or 60% volume fractions into a commercial epoxy-resin matrix and make silk fibre-reinforced epoxy plastic/SFRP. As shown in Figure 2a, the silk fabric is plain woven with two yarns of silk aligned orthogonally. Figure 2b shows the microstructure of a fracture surface of epoxy resin, which features a scale-like morphology. The microstructure of the fabricated composites in Figure 2c reveals the *Ap* silk fibres to be well embedded in the epoxy matrix fabrics. Moreover, using a vacuum treatment on the preform with a relatively high compaction pressure of 0.3 MPa during hot-pressing^39^, the epoxy was well permeated into the gaps between the fibres, although the enlarged image in Figure 2d suggests that the interfacial bonding could be further improved, *e.g*., through surface modification of the silk fibre, as small gaps still remained between the fibre and the matrix. It is also noted that the irregular cross-sectional shape of the silk fibres in forming tightly-packed silk clusters could contribute to their better compaction response, in comparison to that reported for plant fibres^40^. Regarding the heating effect on the properties of silk fibres, the thermal degradation temperature of above 220 °C and the glass transition temperature of 210 °C for *Bm* silk ensure that the silk fibres would not thermally degrade or physically change in structure at 120 °C^41,42^. Thus, it is assumed the fabrication process does not affect the intrinsic mechanical properties of the silk reinforcement.

Typical tensile stress-strain behaviours of the unreinforced epoxy resin and two species of silk composites (60 vol.% *Bm*-SFRP and 50/60 vol.% *Ap*-SFRP) are shown in Figure 2e, and indicate the much improved tensile toughness of the silk composites. The epoxy resin failed in brittle (catastrophic) fashion, whereas all silk-based composites displayed evidence of macroscopic yielding, especially the *Ap*-SFRPs which failed in ductile fashion with a 9% failure strain, far larger than 3% for the pure epoxy resin. As shown in Figure 2g, the 60 vol.% *Bm*-SFRP and 60 vol.% *Ap*-SFRP composites have a specific tensile modulus and a specific tensile strength that are at least twice of that of the unreinforced epoxy resin. Owing to its superior elongation, the breaking energy (calculated as the area under the stress-strain curve) of the 60 vol.% *Ap*-SFRP is 11.7 MPa, *i.e*., an order of magnitude higher than the value of 1.1 MPa for the unreinforced epoxy resin. The fractography in the inset to Figure 2e shows many fractured fibres and holes from pulled-out fibres on the fracture surface for the SFRP, indicating significant contributions from the fracture of the silk fibres and the silk-epoxy interfaces. The flexural behaviour and properties in Figure 2f and h show that the 60 vol.% *Ap*-SFRP has 14% elongation, with a factor of three times larger specific flexural strength and more than ten times larger breaking energy than the unreinforced epoxy resin, clearly demonstrating an even greater strengthening and toughening effect of silk reinforcement under flexural, as opposed to uniaxial tensile, deformation.

In a previous study, we showed that a high-volume fraction, exceeding ~50%, of *Bm* silk reinforcements could significantly elevate the tensile and flexural strength and toughness of silk-epoxy composites^39^. For *Ap*-SFRPs, the fibre volume fraction also has a significant effect on the composite’s mechanical properties (Supplementary Table S1). We found that a brittle-to-ductile transition occurs with a relatively low volume fraction of 30% for *Ap*-SFRPs; specifically, a mere 30 vol.% *Ap* fibres in the composite increased the flexural failure strain from ~4% in the unreinforced epoxy to ~6% in the SFRP, the 50% increase in ductility being sufficient to radically change the mode of failure from brittle to ductile.

### Mechanical properties at sub-ambient temperatures

**Figure 3:**
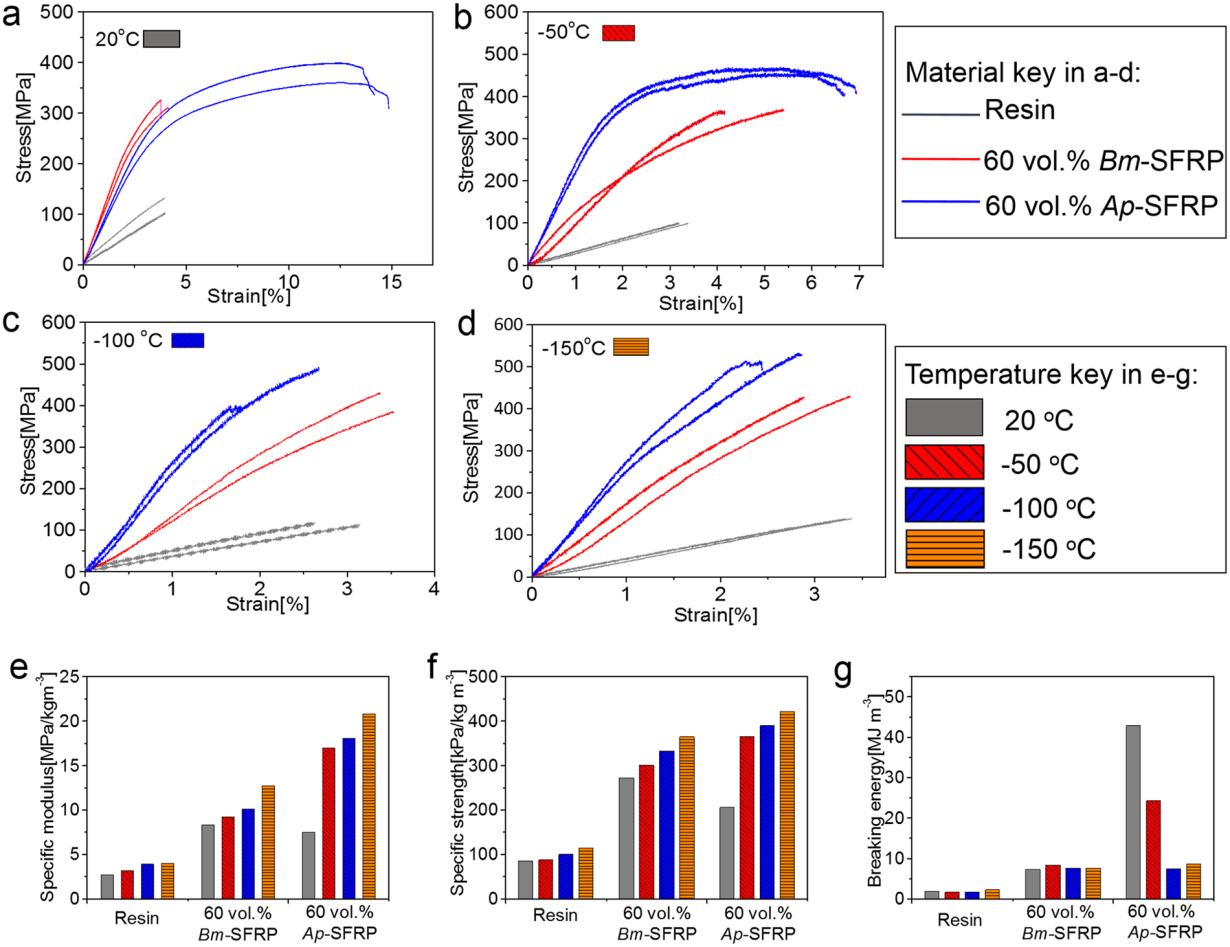
Repeated stress-strain curves of pure epoxy resin and SFRPs from *Bm* and *Ap* silk reinforcements (at quasi-static strain rate under flexural deformation) at temperatures: (**a**) 20 °C; (**b**) −50 °C; (**c**) −100 °C and (**d**) −150 °C. Comparisons of flexural mechanical properties including specific flexural modulus (**e**), specific flexural strength (**f**) and breaking energy (**g**) of pure epoxy resin and SFRPs at the four temperatures.

To further explore the potential of the newly fabricated silk-epoxy composites, especially with regard to their fracture resistance or toughness, we additionally examined the mechanical performance of these composites at sub-ambient temperatures. When the temperature is lowered or the strain rate is increased, structural relaxations in polymeric materials, such as chain segmental movements, often become restricted due to a lack of thermal energy, thereby conferring brittleness to the polymer. Figure 3a–d show the representative flexural stress-strain behaviour of the unreinforced epoxy resin and the *Bm*/*Ap*-SFRPs, respectively at room temperature (20 °C) and at the three sub-ambient temperatures of −50°, −100° and −150 °C. The unreinforced epoxy resin has a fracture strain of 3–4% over the 20° to −150 °C temperature range; its specific initial modulus, specific strength and breaking energy all show a slight increase as the temperature decreases from ambient to −150 °C. Our SFRP composites show a similar trend of increased specific modulus and specific strength with decreasing temperature, but now with enhanced ductilities. Specifically, the *Ap*-SFRP displays distinctly ductile deformation at 20 °C and at −50 °C, with respective elongations of ~14% and ~7%. Figure 3e,f compare the derived flexural mechanical properties at 20 °C, −50 °C, −100 °C and −150 °C, where it can be seen that both the *Bm*-SFRP and *Ap*-SFRP composites, with a fibre volume fraction of 60%, have superior specific properties to the unreinforced resin. Specifically, the *Ap*-SFRP possesses the highest specific modulus and strength at all the three sub-ambient temperatures. At −50 °C, the *Ap*-SFRP has a balanced strength of 471 MPa and a ten-fold increase in breaking energy from 1.7 to 24.3 MJ m^−3^. These findings serve to extend the temperature range for the safe application of epoxy-resin composites to at least −50 °C.

As shown in Supplementary Figure S1, both the *Bm* and *Ap* single fibres, which are taken from the reinforcement silk fabric, show increasing tensile modulus and strength with decreasing temperatures down to −150 °C. Despite the variation in properties between individual fibres, *Ap* silks are found to remain tough even at −150 °C. The plastic strains in *Ap* silk can reach as high 30% at −100 °C and many fibres maintain a more than 10% elongation at stresses that exceed the yield strength at −150 °C. The surprising toughness of silk fibres at sub-ambient temperatures, which has been noted in other studies^43–46^, is strongly confirmed in the present study; we are further reporting that this can significantly contribute to the low-temperature toughness properties of epoxy-resin epoxy composites.

### Impact behaviour

In addition to their low-temperature properties, epoxy-resin composites can also display inadequate fracture resistance under impact loading^47,48^. Here we similarly investigate the use of natural silk fibre reinforcements to potentially alleviate this problem as well. To this end, we apply here a modified Charpy impact test by using unnotched specimens instead of notched for measuring the impact (breaking) strength of our materials. As viewed in Supplementary Movies S1–5, the hammer is dropped from a height of 0.23 m to impact specimens at a speed of 2.9 m s^−1^. Table 1 provides the impact strengths and specific impact strengths for pure epoxy resin and four silk composites. Compared to the unreinforced epoxy resin matrix, the 30 vol.% *Bm*-SFRP displays a ~50% increase in impact strength, whereas 60 vol.% *Bm*-SFRP shows even more dramatic increase of ~450%, compared to that of unreinforced epoxy resin. The *Ap*-SFRP composites are even better with the 30 vol.% *Ap*-SFRP showing 300% increase in impact strength over the unreinforced matrix, and the 60 vol.% *Ap*-SFRP a further factor of three times tougher (estimated to be ~150 kJ m^−2^). A more detailed picture of the trend of increasing impact strength with increasing fiber volume fraction can be seen in Supplementary Table S1. These data clearly show how silk fibre reinforcements can solve the problem of the low impact resistance of epoxy-resin composites, and that higher fibre volume fractions can accentuate this trend. It is worth mentioning that a high volume-fraction carbon fibre reinforced epoxy resin composite (*e.g*., 70 vol.%) only shows impact strength of ~45 kJ m^−2^ under the same conditions, which proves the superiority of SFRP reinforced by silk in impact performance.

**Table 1:** Impact properties of the epoxy resin, *Bm*- and *Ap-*SFRPs with different volume fraction. *σ*_*i*_: impact strength (kJ m^−2^), *σ*_*i*_/*ρ*: specific impact strength (J m^−2^/kg m^−3^).

Sample	Impact strength *σ*_*i*_ kJ m^−2^	Specific Impact Strength *σ*_*i*_/*ρ* J m^−2^/kg m^−3^
Unreinforced Resin	12.8 ± 0.2	10.7 ± 0.1
30 vol.% *Bm*-SFRP	18.5 ± 1.4	15.0 ± 1.2
60 vol.% *Bm*-SFRP	70.7 ± 1.7	56.1 ± 1.4
30 vol.% *Ap*-SFRP	52.5 ± 3.1	42.7 ± 2.5
60 vol.% *Ap*-SFRP	>100	>79.4

**Figure 4:**
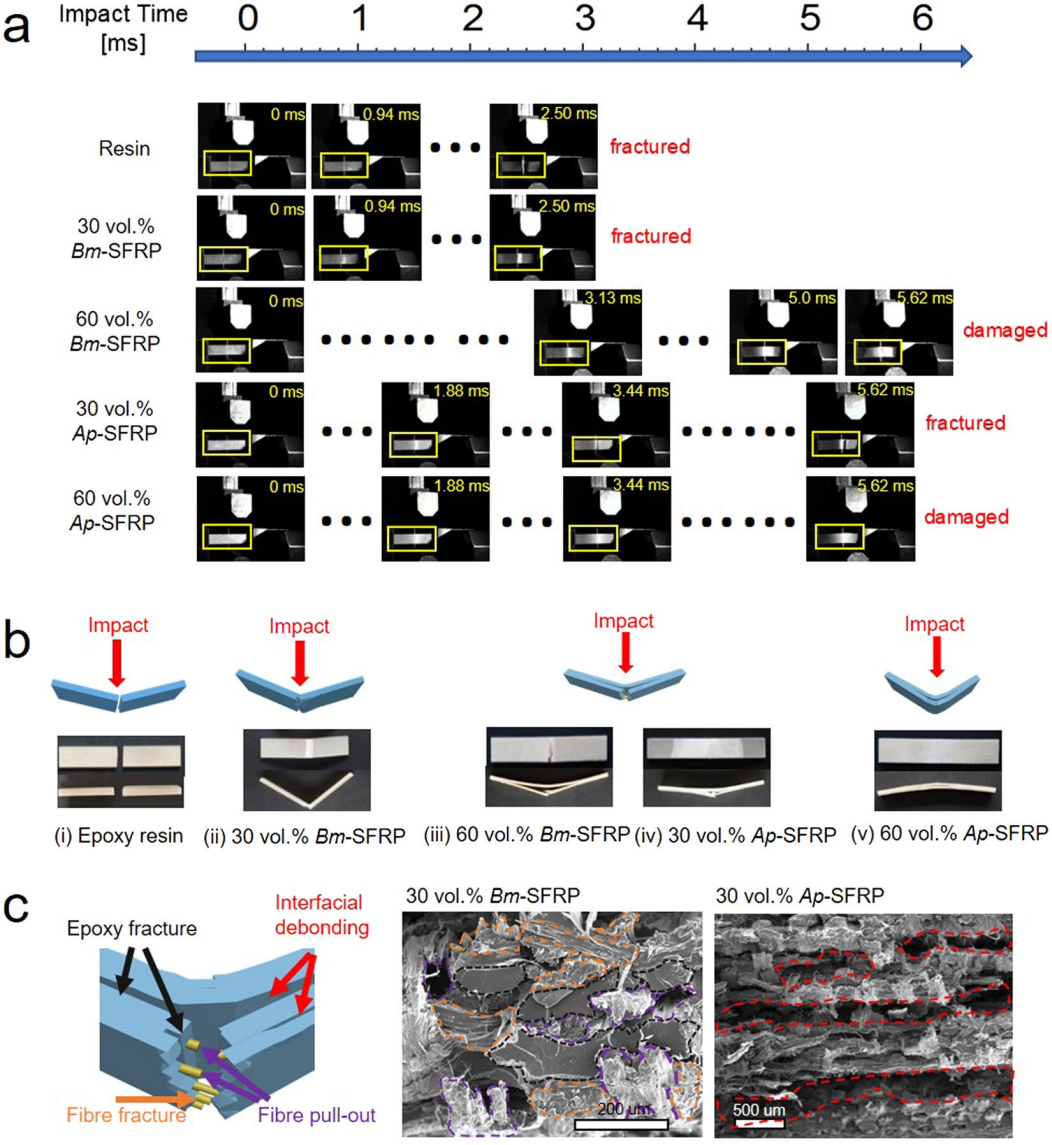
(**a**) High-speed camera images of the impact process of the pure epoxy resin, and *Bm*- and *Ap*-SFRPs with 30 vol.% or 60 vol.% silk reinforcement; (**b**) Schematics of the fracture morphologies after impact tests and photographs of the fractured samples from top and side views. (i) matrix resin’s brittle fracture; (ii) 30 vol.% *Bm*-SFRP’s semi-ductile fracture; ductile fracture of (iii) 60 vol.% *Bm*-SFRP and (iv) 30 vol.% *Ap*-SFRP; (v) ductile response of 60 vol.% *Ap*-SFRP. (**c**) Sketch map of mechanisms for ductile responses to impact on the left, including epoxy resin fracture, fibre pull-out and fracture, interfacial debonding; SEM images on the right showing contributions on a fracture surface: epoxy fracture, fibre fracture and pull-out are highlighted contributions in 30 vol.% *Bm*-SFRP; and interfacial debonding is highlighted as a significant contribution in 30 vol.% *Ap*-SFRP.

From a mechanistic perspective, Figure 4 provides details of the impact process and the fracture morphologies of the silk composites and unreinforced epoxy resin. The high-speed camera recordings in Figure 4a display the critical stages of damage evolution during the impact fracture process on a millisecond scale. The fracture process only lasts for 2.2 ms for unreinforced epoxy resin, whereas this duration is increased to 2.5 ms for the 30 vol.% *Bm*-SFRP and further increases to 5.6 ms for the 60 vol.% *Bm*-SFRP, 30 vol.% *Ap*-SFRP and 60 vol.% *Ap*-SFRP composites. Notably, both 60 vol.% silk composites are only partially damaged and resist complete fracture. It is also noted that as the crack propagates perpendicular to the impact direction, interfacial debonding develops, where fibres are pulled out and openings are generated, as is shown by the whitening of the specimen. Both 60 vol.% silk composites display more interfacial debonding or delamination along the interfaces of the silk fibres, which results in a more effective dissipation of the impact energy.

The various fracture morphologies of the samples are compared in Figure 4b. The unreinforced epoxy resin shows a typical brittle fracture without evidence of much deformation; in contrast, all the silk composites display ample evidence of plastic deformation and extensive subcritical cracking. Moreover, the 30 vol.% *Bm*-SFRP composite shows little to no interfacial debonding prior to fracture, whereas this can be clearly seen in the 60 vol.% *Bm*-SFRP and both *Ap*-SFRP composites. Figure 4c further summarises the main impact energy absorption/dissipation mechanisms for SFRPs, and highlights the contributing mechanisms for composites from the two silk species. For the fracture surface of 30 vol.% *Bm*-SFRP, epoxy resin fracture, fibre pull-out and fracture contribute together to resist impact, whereas interfacial debonding is not observed to contribute equally. On the other hand, interfacial debonding is viewed as a major contribution in the fracture surface of 30 vol.% *Ap*-SFRP.

## Discussion

As demonstrated in other studies^34,43,45,46^, natural silks especially spider dragline silks display enhanced tensile elastic modulus and strength without compromise to the elongation at low temperatures such as −60 °C. Nevertheless, the underline mechanism for low temperature extensibility remains poorly understood. We have shown here in our experiments that the silkworm silk *Ap* silk fibres could still yield and reach a high elongation of 30% at −100 °C, which is well below the temperature ~−70 °C for the relaxation of hydrocarbon-hydrocarbon interactions for carbon-chain polymers such as nylon^43^. The increase in the overall breaking energy of silks at low temperatures down to −100 °C is clearly reversing the trend of decrease in toughness at lower temperatures reported for most polymeric materials. Yet the molecular structure that combines ordered crystalline domains and disordered domains^35^, and the morphology with nano- and micro-fibrillar features^26,29,43^ in natural silks produced from a unique spinning system may be the reason for the surprising low temperature toughness. More specifically, to quantify the change in hydrogen bonding – the most important intermolecular interactions in silk polymers - in both the ordered and disordered domains at low temperatures is more challenging. Despite the difficulty in explaining the low temperature toughness of natural silks, the potential of silk reinforcements for low temperature applications is clearly high. To incorporate silks in composites (*i.e*., in the epoxy resin matrix used here) is significantly one way to improve the low temperature performance of the composite as well as to prevent catastrophic brittle failure. It is also expected that greater low-temperature toughness will be achieved by the finer design of composition and the matrix-fibre interfaces of the silk-epoxy resin composite.

The impact performance of our materials is clearly affected by the impact properties of the individual constituents and the interactions between the fibre and the matrix^49–51^. Here we discuss two main contributions to the enhanced impact toughness, in the form of increased fracture duration and higher capacity for energy absorption, of our silk composites: (i) the greater strength (~2–4 times) and much greater toughness (~20–40 times) of the silk fibres compared to the epoxy resin, and (ii) an appropriate level of interfacial bonding between silk and epoxy-resin matrix, to permit, yet at the same time provide resistance to, fibre pull-out, which is a major toughening mechanism in these materials. The evidence to support the first contribution stems from earlier studies^36,38^, specifically that silk fibres can generate both elastic and plastic deformation to store and dissipate the impact energy during a bullet impact experiment. The many fractured fibres on the fracture surface and the large flexural deformation provide verification of this substantial effect from the elastic-plastic deformation of the silk fibres (Figure 4b,c). For the second contribution, although high interfacial bonding allows efficient stress and energy transfer, our results suggest that too strong an interface may lower the overall energy absorption by inactivating extrinsic toughening mechanisms such as interfacial debonding and fibre pull-out. An indication of this may be that the interlaminar shear strength for the 30 vol.% *Ap*-SFRP is ~23.8 MPa, some 30% lower than that for the 30 vol.% *Bm*-SFRP (~34.0 MPa), yet the impact strength of the *Ap*-SFRP is much larger than that of the *Bm*-SFRP. Recent modelling work on carbon nanomaterials reinforced epoxy composites suggest that simply increasing interfacial strength, or the reinforcement stiffness, will not always confer greater fracture toughness^5,52^. In light of this, there is still a need in future work to seek a deeper understanding of the relationships between fibre, matrix and interface properties and the optimal fracture toughness in silk-epoxy composites.

In summary, we have successfully developed strong and tough epoxy-resin composites through the use of natural silk as a fibre-reinforcement. The mechanical toughness of the silk-epoxy composites is improved significantly at room temperature, sub-ambient temperatures and under impact loading conditions. Natural silk that nicely balances stiffness, strength and toughness clearly differentiates itself from the super-stiff and strong carbon fibres; accordingly, we believe that the extended use of natural silk fibres could inspire novel designs for new high-toughness and light-weight structural composites. Perhaps more importantly, the ductile failure behaviour of silk fibres and other natural systems appears to provide a means of addressing the ever-changing environmental, thermal and mechanical demands of the next generation of engineering synthetic composites.

## Methods

### Materials

The epoxy resin E-51 (bisphenol A type) and the curing agent DS-300G (a modified aromatic amine) were purchased from Dasen Material Science & Technology. Inc. (Tianjin, China). The same epoxy system was applied previously^39^. The basic properties of the cured epoxy resin using a mix ratio of 100:84 can be found in Supplementary Table S2.

Woven *Ap* silk fabric of 0.15 mm thick was purchased from Beijing Rui Fu Xiang Silk Store (Beijing, China), whereas woven *Bm* silk fabric of 0.10 mm thick was purchased from Huzhou Yongrui Textile Co. Ltd. (Zhejiang Province, China).

### Composite fabrication

Layers of silk fabrics were applied uncured epoxy and then laid up. The composite preform was transferred to a hot compression moulding process for 2 hr at 120 °C with a compaction pressure of 300 kPa for consolidation. A vacuum treatment (0.1 kPa for 1 hr) was used to accelerate the infiltration of the epoxy and for air bubble removal prior to hot compression.

A series of *Ap* and *Bm* silk laminates with ~2 mm thickness were prepared with varied volume fractions ranging from 30 vol.% to 60 vol.%. The volume fractions were calculated based on the mass fractions of the silk and the densities of silk and the composite. The density of *Ap* and *Bm* silk was assumed to be 1300 kg m^−3^, as cited in the literature^53^. The density of all the composite specimens was measured via a drainage method using Electronic Density Balance (FA1104J).

### Microstructural and morphological analysis

The microstructures and morphology analysis of the epoxy resin, woven silk fabric and the manufactured *Ap*- and *Bm*-SFRPs were characterized using scanning electron microscopy (SEM, JEOL JSM-6010, Japan) with a 20 kV accelerating voltage in secondary electron imaging mode.

### Quasi-static mechanical testing

Uniaxial tensile testing was performed according to the Chinese Standard GB/T1040-92. Specifically, the tensile mechanical properties were evaluated using an Instron 8801 screw-driven testing machine (Instron Corp., Norwood, MA, USA), at a displacement rate of 2 mm.min^−1^. The flexural testing method and flexural samples were prepared according to the Chinese Standard GB/T1449-2005. Flexural mechanical properties were evaluated on an Instron 5565 screw-driven testing machine (Instron Corp., Norwood, MA, USA), also operating at a displacement rate of 2 mm.min^−1^.

### Mechanical testing at sub ambient temperatures

The tensile properties of the single *Bm* and *Ap* fibres were measured at temperatures of −50 °C, −100 °C and -150 °C under quasi-static load control mode on a dynamic mechanical analyser (TA Instruments, Waters Ltd., DMA Q800). The cross-sectional area measurement was following an established procedure^30^, and reported in SI Figure S2 and the note.

The cryogenic flexural properties of the SFRP composites were measured at temperatures of −50 °C, −100 °C and −150 °C using an Instron 8801 universal tensile tester (with a low-temperature environmental chamber) at a displacement rate of 2 mm.min^−1^. The testing method and samples were also prepared according to the Chinese Standard GB/T1449-2005.

### Impact testing

The impact properties of the composites were measured at room temperature by means of an MTS pendulum impact testing machine (model of ZBC 1000 supplied by MTS Corp., Eden Prairie, MN, USA). The impact tests were conducted according to the ISO179:1997 Standard. Unnotched specimens were loaded flat-wise with a hammer, the mass and the height of which determine the potential energy to be 2 J, and the estimated displacement rate was 3.8 m.s^−1^ (>10^4^ quasi-static displacement rate). A high-speed camera (model of FASTCAM MINI UX100) was used to observe the fracture process during the impact testing.

### Interlaminar shear tests

Interlaminar shear tests were performed on the Instron 5565 screw-driven testing machine at a displacement rate of 1 mm.min^−1^, according to International Standard ISO14130:1997.

### Data availability

Experimental data supporting the findings of this study are available from the corresponding authors upon request.

## Electronic supplementary material

Supplementary Information

Movie S1

Movie S2

Movie S3

Movie S4

Movie S5
