# Supplementary material for: Enhancing the Mechanical Toughness of Epoxy-Resin Composites Using Natural Silk Reinforcements
Source: Sci Rep. 2017 Sep 20;7:11939. doi: 10.1038/s41598-017-11919-1 (PMC5607334; doi:10.1038/s41598-017-11919-1)
Supplement: Supplementary file 1 — Supplementary Information [file 41598_2017_11919_MOESM1_ESM.pdf]

Supplementary Information

Enhancing the Mechanical Toughness of Epoxy-Resin Composites Using Natural Silk Reinforcements

Kang Yang[1], Sujun Wu[1], Juan Guan[1]*, Zhengzhong Shao[2], Robert O. Ritchie[1,3]*

[1] Intl. Research Center for Advanced Structural and Biomaterials, School of Materials Science and Engineering, Beihang University, Beijing 100191, China.
[2] State Key Laboratory of Molecular Engineering of Polymers, Laboratory of Advanced Materials, Department of Macromolecular Science, Fudan University,Shanghai 200433, China.
[3] Materials Sciences Division, Lawrence Berkeley National Laboratory, Department of Materials Science & Engineering, University of California, Berkeley CA94720, USA.
Correspondence: J.G.(e-mail: juan.guan@buaa.edu.cn) or to R.O.R.(e-mail: roritchie@lbl.gov).

This file contains 2 figures, 2 tables and the list of captions for 5 movies.

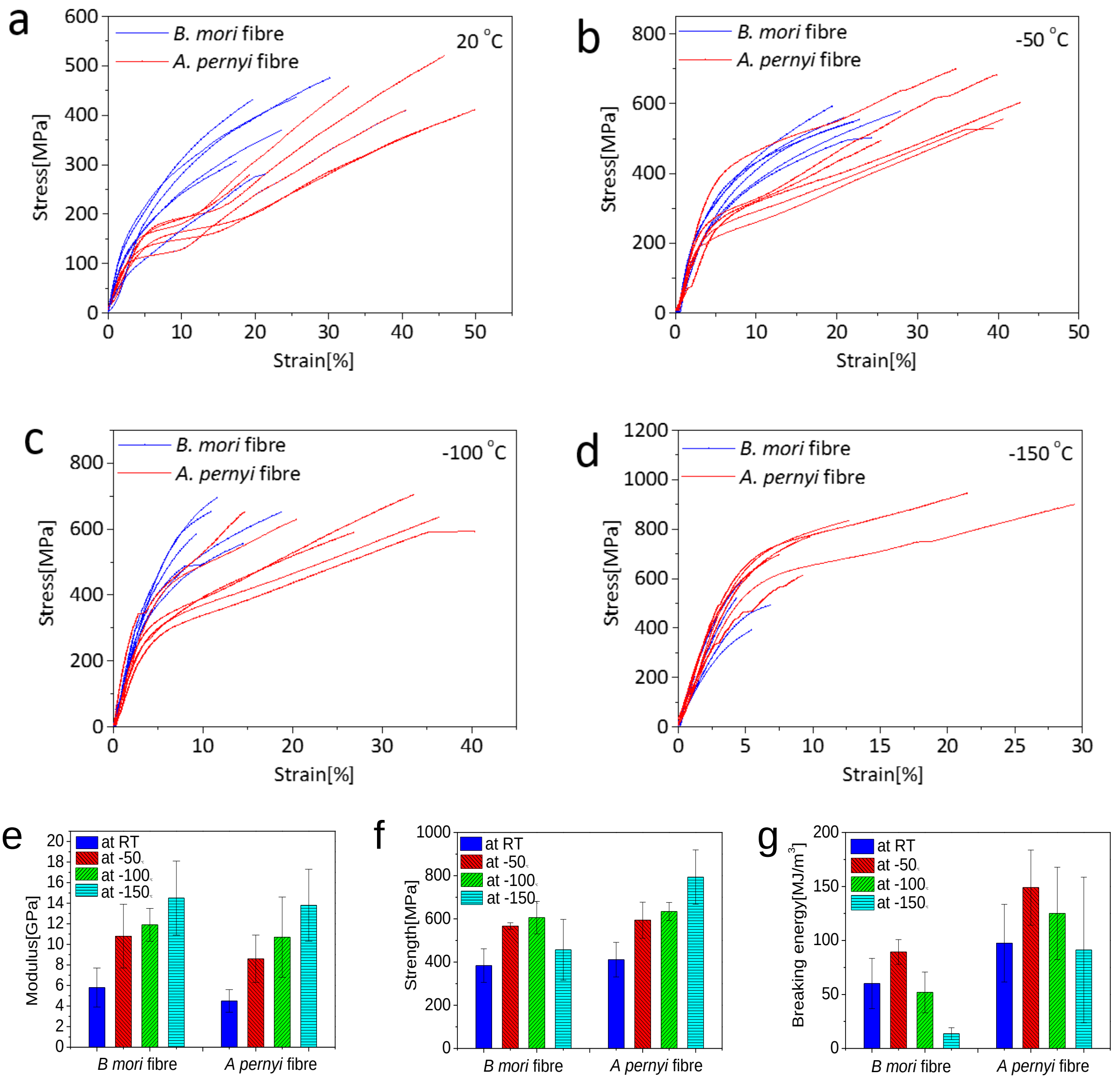

Figure S1. Tensile stress-strain curves of *B. mori* and *A. pernyi* single silk fibres at various temperatures: 20°C (a); -50°C (b); -100°C (c) and -150°C (d); and tensile mechanical properties of *B. mori* and *A. pernyi* silk fibres at various temperatures, RT for room temperature 20°C, -50°C, -100°C and -150°C: Initial tensile modulus (e); maximum tensile stress / strength (f); breaking energy (g), which is calculated from the area under the stress-strain curve. Error bars represent standard deviation SD of the mean values.

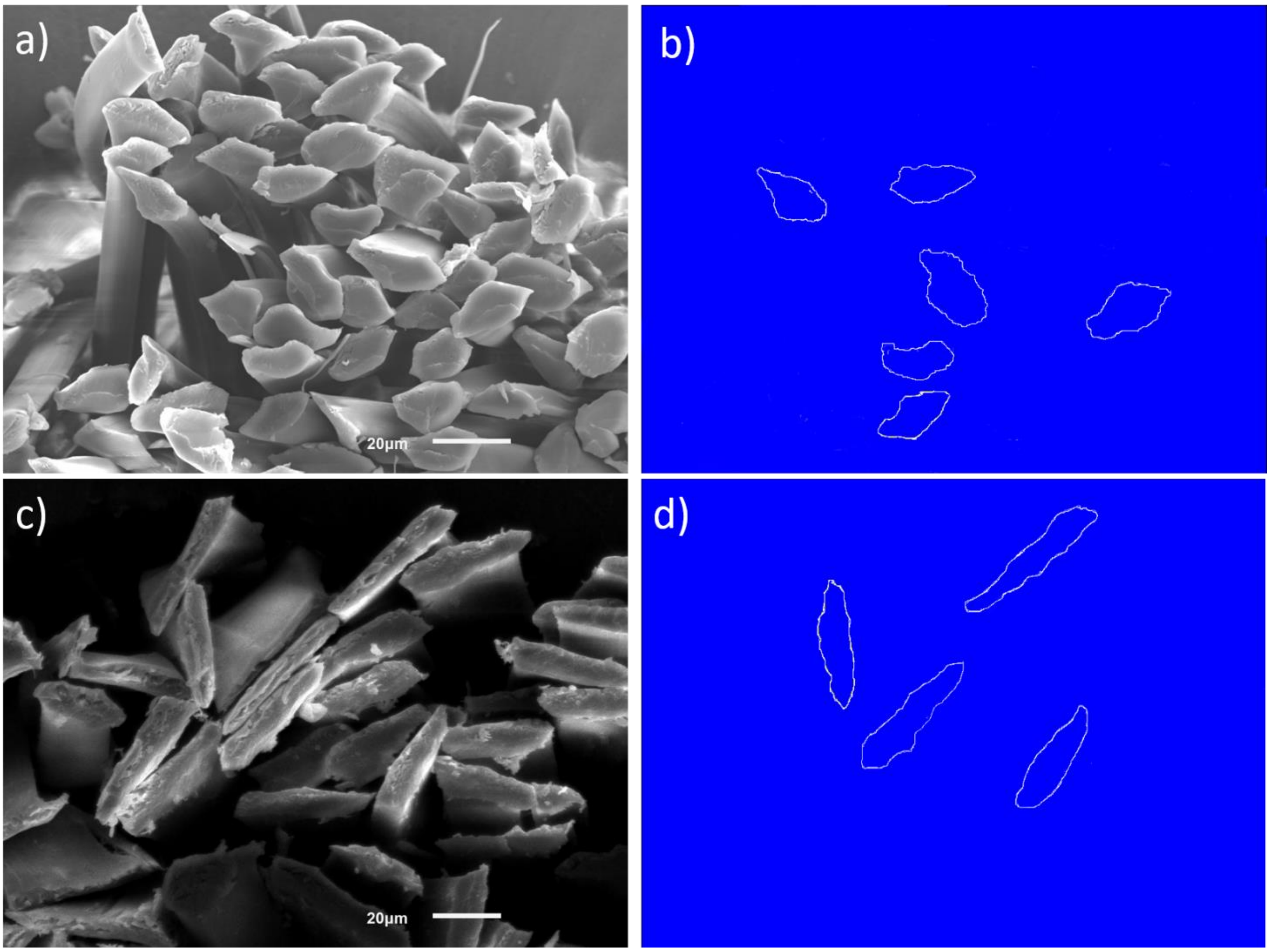

Figure S2. (Left) Cross-sectional images from SEM of silk fibres taken from the fabrics and (Right) Area measurement images from the left-side images using ImageJ software. *B. mori* silk fibres area shown in (a) and (b), and *A. pernyi* silk fibres are shown in (c) and (d).

Note: The cross-sectional areas of silk fibres were measured using the following procedure as reported in ref. [1]. A bundle of silk fibres, carefully taken from the fabric, were pulled through a plastic tube as the fibres needed to tightly pack together, short sections of the tube were cut perpendicularly to obtain cross-sections of the silk fibres, and finally the fibres cross-section were imaged in the scanning electron microscope for photo acquisition and area measurement using ImageJ software. The measured average areas were 151 μm^2 for *B. mori* silk and 291 μm^2 for *A. pernyi* silk.

Reference

[1] Guan J, Porter D, Vollrath F. Silks cope with stress by tuning their mechanical properties under load. *Polymer* **53**, 2717-2726 (2012).

Table S1. Mechanical properties of epoxy resin and the *Ap*-SFRPs.

Specimen	Tensile properties						Flexural properties						Impact properties	
	E_t	E_t/ρ	σ_t	σ_t/ρ	ε_t	BE_t	E_f	E_f/ρ	σ_f	σ_f/ρ	ε_f	BE_f	σ_i	σ_i/ρ
**Epoxy resin**	3.2±0.1	2.7±0.1	76.6±1.3	63.8±1.1	2.8±0.1	1.1±0.1	3.5±0.0	2.9±0.0	134.2±6.4	111.8±5.3	3.9±0.1	3.0±0.2	12.8±0.2	10.7±0.1
**30 vol.%-*Ap***	-	-	-	-	-	-	5.0±0.2	4.1±0.2	146.9±9.1	119.4±7.4	5.8±0.2	5.5±0.2	52.5±3.1	42.7±2.5
**40 vol.%- *Ap***	-	-	-	-	-	-	6.5±0.3	5.2±0.2	238.9±13.3	192.7±10.7	7.7±0.1	14.4±0.6	62.3±5.8	50.2±4.7
**50 vol.%- *Ap***	7.8±0.2	6.2±0.2	129.3±3.2	103.4±2.6	9.6±0.1	9.9±0.3	6.8±0.3	5.4±0.2	256.6±13.6	205.3±10.9	11.9±0.0	24.5±1.1	90.8±5.4	72.6±4.3
**60 vol.%- *Ap***	8.1±0.2	6.4±0.2	160.3±3.1	127.2±2.5	8.9±0.1	11.7±0.2	9.4±0.3	7.5±0.2	398.6±16.4	316.3±13.0	13.2±0.1	42.3±1.5	＞100	＞79.4

E_t: tensile modulus(GPa), ρ:density (kg m^{-3}), E_t/ρ: specific tensile modulus (MPa/kg m^{-3}), σ_t: tensile strength (MPa), σ_t/ρ: specific tensile strength (kPa/kg m^{-3}), ε_t: ultimate tensile strain (%), BE_t: tensile fracture energy(MJ m^{-3}), E_f: flexural modulus (GPa), ρ: density(kg m^{-3}), E_f/ρ: specific flexural modulus (MPa/kg m^{-3}), σ_f: flexural strength (MPa), σ_f/ρ: specific flexural strength (kPa/kg m^{-3}), ε_f: ultimate flexural strain (%), BE_f: flexural fracture energy (MJ m^{-3}), σ_i: impact strength (kJ m^{-2}), σ_i/ρ: specific impact strength (J m^{-2}/kg m^{-3})

Table S2. Basic physical and mechanical properties of the epoxy resin E51 cured by DS-300G.

Physical properties		Mechanical properties			
**Density (kg·cm^{-3})**	**Viscosity (mPa·s)**	**Tensile modulus (GPa)**	**Tensile strength (MPa)**	**Ultimate strain (%)**	**Impact strength (kJ·m^{-2})**
1200	40	3.0-3.2	73-78	2.7-2.9	12.5-13.0

List of Supplementary Movies:

Movie S1. Impact behaviour of unreinforced epoxy resin from 3 specimens.
Movie S2. Impact behaviour of 30 vol.% *Bm*-SFRP from 3 specimens.
Movie S3. Impact behaviour of 60 vol.% *Bm*-SFRP from 3 specimens.
Movie S4. Impact behaviour of 30 vol.% *Ap*-SFRP from 3 specimens.
Movie S5. Impact behaviour of 60 vol.% *Ap*-SFRP from 3 specimens.
